# Small RNAs in the pathogenesis of preeclampsia

**DOI:** 10.1016/j.placenta.2024.06.009

**Published:** 2024-06-17

**Authors:** William R Cooke, Gabriel Davis Jones, Christopher WG Redman, Manu Vatish

**Affiliations:** a Nuffield Department of Women’s and Reproductive Health, University of Oxford, Level 3 Women’s Centre, John Radcliffe Hospital, Oxford, OX3 9DU

**Keywords:** Preeclampsia, Small RNA, Micro RNA, Transfer RNA, Small nucleolar RNA, Piwi-interacting RNA

## Abstract

Preeclampsia is a major contributor to maternal and fetal morbidity and mortality. The disorder can be classified into early- and late-onset subtypes, both of which evolve in two stages. The first stage comprises the development of pre-clinical, utero-placental malperfusion. Early and late utero-placental malperfusion have different causes and time courses. Early-onset preeclampsia (20% of cases) is driven by dysfunctional placentation in the first half of pregnancy. In late-onset preeclampsia (80% of cases), malperfusion is a consequence of placental compression within the confines of a limited uterine cavity. In both subtypes, the malperfused placenta releases stress signals into the maternal circulation. These stress signals trigger onset of the clinical syndrome (the second stage). Small RNA molecules, which are implicated in cellular stress responses in general, may be involved at different stages. Micro RNAs contribute to abnormal trophoblast invasion, immune dysregulation, angiogenic imbalance, and syncytiotrophoblast-derived extracellular vesicle signalling in preeclampsia. Transfer RNA fragments are placental signals known to be specifically involved in cell stress responses. Disorder-specific differences in small nucleolar RNAs and piwi-interacting RNAs have also been reported. Here, we summarise key small RNA advances in preeclampsia pathogenesis. We propose that existing small RNA classifications are unhelpful and that non-biased assessment of RNA expression, incorporation of non-annotated molecules and consideration of chemical modifications to RNAs may be important in elucidating preeclampsia pathogenesis.

## Abbreviations

MSC: Mesenchymal stem cells
DNA: Deoxyribonucleic acid
mRNA: Messenger RNA
miRNA: Micro RNA
piRNA: Piwi-interacting RNA
PlGF: Placental growth factor
RNA: Ribonucleic Acid
sFlt-1: Soluble fms-like tyrosine kinase 1
snRNA: Small nuclear RNA
snoRNA: Small nucleolar RNA
STB-EV: Syncytiotrophoblast-derived extracellular vesicle
tRNA: Transfer RNA
VEGF: Vascular endothelial growth factor

## 1 Preeclampsia disease burden

Preeclampsia is a complex, progressive disorder of pregnancy, characterised by maternal hypertension with multi-organ involvement [1]. With an estimated global prevalence of 4.6%, preeclampsia disproportionately impacts those in lower-income regions [2], accounting for 14% of maternal deaths worldwide [3]. This translates to an estimated 50-100 women suffering substantial morbidity for every maternal mortality [4]. Preeclampsia also represents a major burden for the fetus. Globally, it accounts for approximately 500 000 perinatal deaths annually [5]. Even in higher-income regions, the impact of perinatal morbidity is important; 18% of iatrogenic preterm births were attributed to preeclampsia in England between 2015 and 2017 [6].

Clinical interventions to reduce the impact of preeclampsia are limited, aside from delivery, which begins the process of resolution. Aspirin is used for preeclampsia prophylaxis, despite varying evidence concerning efficacy and no consensus on its mechanism of action [7]. Angiogenic biomarkers have proven capable of ruling out preterm preeclampsia; however at present there are no biomarkers for the successful diagnosis of preeclampsia [8]. After clinical diagnosis of the disorder, management is supportive and focussed on timing delivery of the fetus to minimise pregnancy complications. There remain no disease-modifying interventions. Improvements to our understanding of the molecular mechanisms underlying the disorder could help direct future clinical care.

## 2 Preeclampsia subclassification and pathophysiology

Preeclampsia can be subclassified by gestational age at onset, distinguishing cases with early-onset (<34 weeks of gestation) from those with late-onset (≥34 weeks of gestation) [9]. This distinction reflects differences in both clinical phenotype and molecular associations. Both subtypes can be considered to evolve in two stages (Figure). This model was first described in 1991 [10] but is still considered the most effective paradigm to frame the biological mechanisms underlying preeclampsia [4,11]. The first stage comprises the development of asymptomatic uteroplacental malperfusion. The malperfused placenta releases stress signals into the maternal circulation, which then cause the second symptomatic stage of the syndrome. Early and late utero-placental malperfusion have very different causes.

Early-onset preeclampsia (20% of cases) is driven by dysfunctional placentation in the first half of pregnancy. In healthy pregnancy, the spiral arteries lose their musculo-elastic walls and are remodelled into dilated non-contractile arteries that transmit blood at high volume and low velocity into the intervillous space [12]. In early-onset preeclampsia, deficient spiral artery remodelling results from immune interactions between decidual uterine natural killer cells (maternal) and HLA-C expressing extravillous trophoblast cells (fetal) during the first trimester [13]. The earlier onset results in fetal growth restriction and the need for preterm delivery.

In contrast, the first stage of late-onset preeclampsia (80% of cases) is common to all pregnancies around term. It results from continuing placental growth in a confined uterine space. The intervillous space is compressed as the chorionic villi are packed together more closely. Oxygen content of intervillous blood progressively falls. Feto-placental hypoxic stress is then associated with increased rates of fetal growth restriction, preeclampsia or stillbirth at or beyond term (the post-mature syndromes) [14].

The maternal syndrome in the second stage is characterised by vascular inflammation, with diffuse endothelial activation [15,16]. This is manifest in the kidney as glomerular endotheliosis, a characteristic lesion of preeclampsia comprising endothelial cell swelling and loss of endothelial cell fenestrae causing proteinuria [17]. Hepatic inflammation can lead to microthrombosis, necrosis and “sinusoidal obstruction syndrome” [18]. Cerebral oedema is a rare complication associated with neuroinflammation and breakdown of the blood brain barrier [19]. Activated endothelium is procoagulant, increasing platelet consumption. Impaired release of nitric oxide from the dysfunctional endothelium reduces the vasodilatory signal to adjacent vascular smooth muscle cells contributing to hypertension [20].

Syncytiotrophoblast stress is the feature common to early- and late-onset preeclampsia. Stress signals, released into the maternal circulation, promote the maternal syndrome. The best characterised is soluble fms-like tyrosine kinase 1 (sFlt-1), mainly produced by the syncytiotrophoblast [21]. There are many others, including syncytiotrophoblast-derived extracellular vesicles [22]. The time course of changes in sFlt-1 levels in healthy pregnancies indicates that syncytiotrophoblast stress begins on average around 32 weeks of gestation and progresses to term and beyond [23]. At term, syncytiotrophoblast displays increasing apoptosis, autophagy, syncytial knots and necrosis; all ascribable to stress. However, delivery (spontaneous or induced) usually pre-empts pathological outcomes. Preeclampsia may arise from different pathways to this common endpoint, influenced by maternal genetics, epigenetics, lifestyle and environment factors with different fetal and maternal responses to the ensuing insults. This is reflected in the clinical heterogeneity of the disorder.

Small non-coding RNAs are increasingly recognised as contributors to these pathogenic pathways, through regulation of early trophoblast invasion and as syncytiotrophoblast stress signals, amongst which tRNA fragments are increasingly important [24,25].

## 3 Small RNAs

RNAs are polymers consisting of four nucleotides: adenine, guanine, cytosine and uracil. Typically single-stranded, RNA molecules are synthesised with a sequence complementary to their DNA template, during the transcription reaction. RNAs can be subclassified by size. Different thresholds are used to delineate small RNAs, varying from <50 nucleotides to <300 nucleotides. Often, the threshold is driven by both read length in the RNA sequencing technique used to detect the molecules, and the size selection strategies used to exclude longer RNAs.

Small RNAs can be subclassified by function. Micro RNAs (miRNAs) are 21-23 nucleotide molecules, which silence messenger RNAs (mRNAs) by binding and cleaving them. Transfer RNAs (tRNAs) are approximately 74 nucleotides in length and shuttle amino acids to the elongating peptide during the translation reaction. tRNA fragments (tRFs) are derived by tRNA cleavage and have been identified as pleiotropic regulators of cellular function. Piwi-interacting RNAs (piRNAs; 26-31 nucleotides) are involved in RNA silencing and epigenetic regulation. Small nucleolar RNAs (snoRNAs) are 60-300 nucleotides in length and mediate RNA modifications such as methylation and pseudouridylation. A varying proportion of small RNAs remain unannotated and could yet prove to be functional.

## 4 Relevance of small RNAs to preeclampsia

Studies investigating small RNAs in preeclampsia have compared RNA expression between case and control cohorts. This is an important first step in identifying potential biomarkers implicated in pathogenesis. Experimental models are typically required to demonstrate a causal role for putative small RNA targets in preeclampsia. These experiments often add or block target RNA molecules to elucidate how perturbations may alter function.

Evidence for small RNAs in preeclampsia pathogenesis has been found at several stages in the natural progression of the disease: from trophoblast invasion, through angiogenic and immune imbalance to syncytiotrophoblast extracellular vesicle (STB-EV) release and endothelial cell dysfunction. This review describes the key advances and emerging evidence for the lesser-studied small RNA species (summary in Table).

### 4.1 miRNA in preeclampsia

Previous studies have identified up 127 differentially expressed miRNAs in preeclampsia, from varying biological samples and cell types, which have been comprehensively studied and reviewed previously [26–29]. A proportion of these miRNAs have been further investigated, yielding evidence to suggest a functional role in pathogenesis. Here we present preeclampsia-dysregulated miRNAs where evidence suggests a pathogenic link to the disorder, stratified by the preeclampsia pathologies to which the miRNAs contribute.

#### 4.1.1 Trophoblast invasion

The early human placenta in preeclampsia is inaccessible: the disorder is defined by onset after 20 weeks of gestation [1] and placental sampling would end the pregnancy. An alternative strategy to assess small RNA signalling in early human placental development is to combine data from first trimester blood samples with appropriate experimental models, such as first trimester cell lines (e.g. HTR-8/SVneo) or explants.

Fu and colleagues demonstrated downregulation of miR-376c in first trimester plasma from pregnancies which subsequently developed preeclampsia, correlating this with term placental tissue [30]. Inhibition of miR-376c in both HTR-8/SVneo cells and first trimester explants impaired trophoblast invasion and extravillous trophoblast outgrowth. Trophoblast invasion is considered a trigger for spiral artery remodelling during placentation [12]. Further experimental data suggests miR-376c acts by impairing TGF-B/Nodal signalling in trophoblast cells; dysregulation of both proteins has previously been associated with preeclampsia [31]. Together these observations implicate miR-376c as a possible contributor to impaired placentation in early-onset preeclampsia.

miR-210, the best studied miRNA in preeclampsia, is also implicated in abnormal trophoblast invasion. Anton *et al*. identified increased miR-210 expression in second trimester serum from women who would later develop hypertensive disorders of pregnancy [32]. They overexpressed miR-210 in extravillous trophoblasts isolated from first-trimester tissue, finding impaired invasion. Similar effects were seen with ectopic expression of miR-210 in first trimester cytotrophoblast cells [33]. Ephrin-A3 and homeobox-A9, predicted targets of miR-210, were downregulated in placentas obtained at delivery from women with preeclampsia, though the gestational age of these samples was not specified [33]. These findings suggest a second miRNA signalling pathway may be involved in the abnormal trophoblast invasion seen early in this disorder.

#### 4.1.2 Placental angiogenesis

Escudero *et al*. summarised the role of miRNAs on dysfunctional angiogenesis in preeclampsia [34]. miR-126 is highly expressed in human endothelial cells and plays a key role in vascularisation outside pregnancy. Yan *et al*. found that miR-126 was down-regulated in endothelial progenitor cells from preeclampsia placentas. Inhibition of miR-126 reduced endothelial progenitor cell proliferation, migration and increased expression of the anti-angiogenic gene PIK3R2 [35]. These findings imply reduced miR-126 may impair placental angiogenesis. Another group found miRNAs from the miR17-92 cluster were increased in preeclampsia placentas [36]. Computational and transfection studies suggested these miRNAs may target several angiogenesis-related genes including HIF-1A, IL-8, EPHB4, EFNB2 and TIMP2. This implicates the miR17-92 cluster in abnormal angiogenesis in the preeclampsia placenta.

#### 4.1.3 Decidual mesenchymal stem cells

Mesenchymal stem cells (MSCs) regulate proliferation, maturation and function of local immune cells [37]. MSCs from the decidua exhibit altered cytokine expression in preeclampsia and are hypothesised to contribute to the immune imbalance which contributes to the disorder [38]. miR-494 is upregulated in preeclampsia decidual MSCs, compared to normotensive controls. Overexpression of miR-494 in decidual MSCs reduced cell viability and proliferation, as well as VEGF release. Conditioned media from both preeclampsia decidual MSCs and miR-494 overexpressed decidual MSCs impaired HTR-8/SVneo migration. In a separate study, miR-494 overexpressed decidual MSCs were seen to inhibit M2 macrophage polarisation [39]. Thus miR-494 expression in decidual MSCs may regulate immune cells at the materno-fetal interface in preeclampsia.

#### 4.1.4 Angiogenic factors

Soluble fms-like tyrosine kinase-1 (sFlt-1) is increased, and placental growth factor (PlGF) decreased, in the circulation of women with preeclampsia [23]. sFlt-1 is released by the placenta in response to various stressors including hypoxia, and accounts for maternal features of preeclampsia. In particular it sensitises endothelial cells to inflammatory cytokines [40]. Plasma miR-195-5p levels correlate with sFlt-1 concentration in women with preeclampsia [41]. Moreover, human umbilical vein endothelial cells incubated with preeclampsia plasma demonstrate higher miR-195-5p expression and reduced vascular endothelial growth factor expression [42]. Whilst these findings do not demonstrate causality, prior work showing the anti-angiogenic role of miR-195-5p confirms this molecule may play a role in the angiogenic imbalance of preeclampsia [43].

#### 4.1.5 Syncytiotrophoblast-derived extracellular vesicles

Several studies demonstrate differential expression of miRNAs in STB-EVs from women with preeclampsia [44–46]. Cronqvist *et al*. used gold-labelling to demonstrate transfer of functional miRNAs from STB-EVs to the endoplasmic reticulum of endothelial cells in culture, with consequent changes in expression of the FLT1 gene [47]. Shen and colleagues demonstrated increased miR-155 expression in exosomes isolated from preeclampsia serum [44]. Both preeclampsia exosomes and miR-155 overexpressed exosomes inhibited expression of endothelial nitric oxide synthase when added to cultured endothelial cells. Whilst the exosomes were derived from serum without immunoprecipitation, their strong expression of placental alkaline phosphatase (a placentally enriched isoform) suggests syncytiotrophoblast origin. These data suggest a direct link between placenta and maternal hypertension might be mediated by aberrant miRNA expression.

### 4.2 tRNA fragments in preeclampsia

We first reported tRFs in human pregnancy in 2019, when we found them to be the most abundant small RNA species in placental perfusion-derived STB-EVs [48]. Subsequently, we compared expression of small RNA in perfusion-derived STB-EVs from early-onset preeclampsia placentas and normotensive controls, finding again that tRFs accounted for the majority of dysregulated species [49]. Over 900 fragments were differentially expressed in preeclampsia perfusion STB-EVs and upregulation of the most abundant (5’-tRF-Glu-CTC) was corroborated in STB-EVs derived from maternal preeclampsia plasma. 5’-tRF-Glu-CTC triggered a pro-inflammatory response in cultured macrophages, which indirectly activated endothelial cells. Collectively these observations implicate tRFs as markers of syncytiotrophoblast stress in the first stage of preeclampsia, which may signal to relevant target maternal cells to contribute to the second clinical stage of preeclampsia.

One other study has investigated small RNA released by the syncytiotrophoblast, but in larger particles than STB-EVs derived from placental explant cultures [46]. Whilst not a directly comparable experimental design, this study also reported abundant tRFs within the trophoblast “debris” as well as differential tRF expression between preeclampsia and controls. These data corroborate our findings, providing a strong rationale for further investigation of tRFs in relation to preeclampsia pathogenesis.

### 4.3 Small nucleolar RNAs in preeclampsia

Global RNA profiling of early-onset preeclampsia trophoblasts identified differences in snoRNA expression when compared to gestation-matched cells [50]. Nine snoRNAs were differentially expressed, compared to five miRNAs and 303 mRNAs. snoRNAs guide RNA modifications in structures called Cajal bodies within the nucleus. To corroborate these findings, the authors visualised Cajal bodies in preeclampsia and normotensive cytotrophoblasts, finding double the number in second trimester preeclampsia cells over controls. Notably, the control placentas in this study were derived from normotensive spontaneous preterm births, so differences may not be specific to preeclampsia. snoRNAs regulate chemical modifications of RNAs. Some of these modifications limit our ability to detect RNA with conventional approaches [51]. To include this hidden layer of the transcriptome, future studies in this field should consider strategies to account for RNA modifications. Further data regarding snoRNA expression may direct these approaches.

### 4.4 piRNA in preeclampsia

Three studies report expression of piRNAs in relation to preeclampsia. The first assessed expression of piRNAs in 82 placentas from preeclampsia case-control pairs [52]. No significant differences in piRNA expression were identified. Two subsequent studies used smaller samples (≤5 cases), identifying 77 and 148 differentially expressed piRNAs in preeclampsia [53,54]. No piRNAs were overlapping between the two studies and the disagreement between these smaller studies and the larger one (showing no effect) could be a consequence of sampling bias.

Both smaller studies went on to assess expression of piwi-binding proteins, to which piRNA must bind to degrade mRNA. Again, data were not concordant, with one study demonstrating differential expression of PIWIL1 protein in preeclampsia placentas, where the other reported PIWIL1 mRNA to be around the lower limit of detection in placental tissue using qPCR. This study found no differences between preeclampsia and normal PIWIL1 expression. The first study went on to confirm PIWIL1 expression was lower in preeclampsia villous trophoblast cells through immunohistochemistry and Western blotting. Over-expression of PIWIL1 in HTR-8/SVneo cells (a first trimester trophoblast cell line) promoted cell proliferation and invasion. Silencing PIWIL1 had the opposite effect. These data could link differential expression of piRNA to a pathway which may underlie abnormal trophoblast invasion in early-onset preeclampsia. However, a discovery made in a term placenta is being extrapolated to a first-trimester cell line and pathology. Moreover, findings are based on a small sample size and directly contradicted by a similar study from another group. No authors have yet investigated expression of piRNA in syncytiotrophoblast extracellular vesicles, peripheral blood or endothelial cells in preeclampsia.

## 5 Concluding remarks

The domain of small RNA biology is growing. New RNA classes are being investigated and their relevance to preeclampsia pathophysiology uncovered. There are limitations to existing classifications and approaches in this field. Several authors studying small RNAs in preeclampsia report finding a substantial number of unannotated reads with biological importance [49,52,54]. Others have described reads which map to multiple small RNA classes, creating confusion about their biogenesis and function [55]. Some small RNAs which map to one class exhibit actions which are more closely aligned to a different class [56]. These observations suggest that over-reliance on annotation could easily stifle new discoveries.

Conventional small RNA sequencing analysis involves first trimming adapters and removing low quality reads. Reads are then aligned to a series of reference databases, such as miRbase and piRBase for miRNA and piRNA respectively. Reads are annotated, features counted and a differential expression analysis between different conditions is performed. This annotation-first approach has significant shortcomings: unmapped sequences are discarded and multimapping reads are not identified as such. We promote a first-principles approach to small RNA sequencing analysis, such as that employed by the DEUS package [57]. DEUS uses actual read sequences without annotation to determine expression intensities, allowing users to cluster or annotate downstream.

Knowledge in parallel disciplines such as immunology and cancer biology has recently advanced significantly through investigation of small RNAs [58,59]. Small RNA-based therapies have been licensed in the US and Europe, gaining funding and clinician acceptance [60]. We propose that a better understanding of small RNA biology in preeclampsia may ultimately lead to clinically relevant discoveries.

## Figures and Tables

**Figure:**
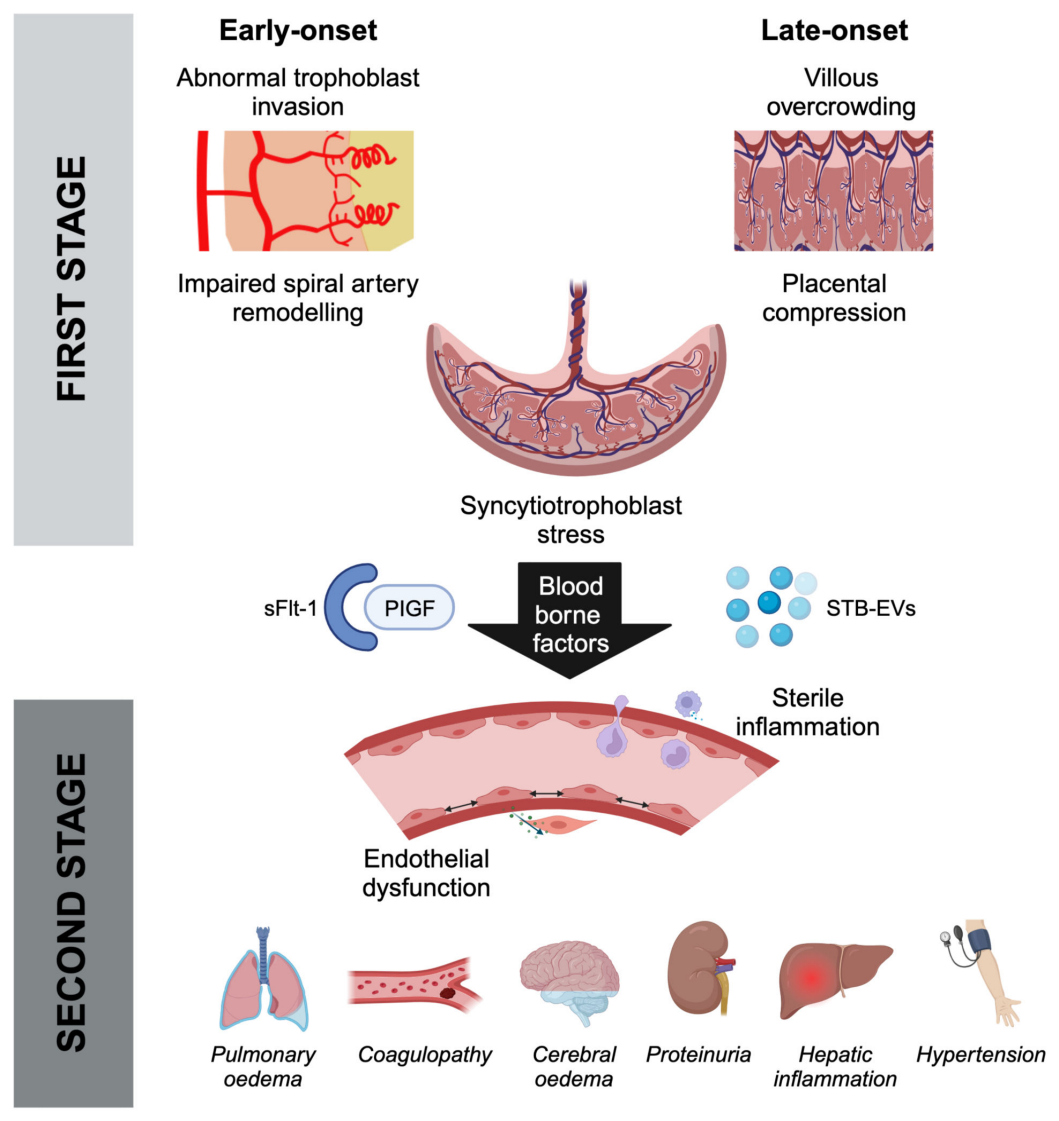
Schematic diagram illustrating the two-stage model of preeclampsia. Created with biorender.com under licence.

**Table:** Summary of small RNAs implicated in the pathogenesis of preeclampsia, presented by class.

Class	Abbreviation	Length (nucleotides)	Proposed role	Implicated molecules	References
Micro RNA	miRNA	21-23	Impaired trophoblast invasion	miR-376c	[30,31]
				miR-210	[32,33]
			Impaired placental angiogenesis	miR-126	[35]
				miR17-92 cluster	[36]
			Decidual mesenchymal stem cell dysfunction	miR-494	[38,39]
			Angiogenic imbalance	miR-195-5p	[41,42]
			Syncytiotrophoblast-derived extracellular vesicle signalling to endothelium	miR-517a, miR-517c, miR-519a, miR-210	[47]
				miR-155	[44]
Transfer RNA fragments	tRF	<74	Endothelial dysfunction via syncytiotrophoblast-derived extracellular vesicle	5’-tRF-Glu- CTC	[49]
Small nucleolar RNA	snoRNA	60-300	Increased Cajal body foci in cytotrophoblasts	-	[50]
Piwi-interacting RNA	piRNA	26-31	Possible impaired villous trophoblast invasion	piR-hsa- 1256314	[54]
